# Lactic Acid Bacteria as Potential Agents for Biocontrol of Aflatoxigenic and Ochratoxigenic Fungi

**DOI:** 10.3390/toxins14110807

**Published:** 2022-11-19

**Authors:** Eva María Mateo, Andrea Tarazona, Misericordia Jiménez, Fernando Mateo

**Affiliations:** 1 Departamento de Microbiología y Ecología, Facultad de Medicina y Odontología, Universitat de Valencia, E-46100 Burjasot, Valencia, Spain; 2 Departamento de Microbiología y Ecología, Facultad de Ciencias Biológicas, Universitat de Valencia, E-46100 Burjasot, Valencia, Spain; 3 Departamento de Ingeniería Electrónica, ETSE, Universitat de Valencia, E-46100 Burjasot, Valencia, Spain

**Keywords:** lactic acid bacteria, biocontrol, *Aspergillus* spp., *Penicillium verrucosum*, aflatoxins, ochratoxin A, fungal growth, machine learning

## Abstract

Aflatoxins (AF) and ochratoxin A (OTA) are fungal metabolites that have carcinogenic, teratogenic, embryotoxic, genotoxic, neurotoxic, and immunosuppressive effects in humans and animals. The increased consumption of plant-based foods and environmental conditions associated with climate change have intensified the risk of mycotoxin intoxication. This study aimed to investigate the abilities of eleven selected LAB strains to reduce/inhibit the growth of *Aspergillus flavus*, *Aspergillus parasiticus*, *Aspergillus carbonarius*, *Aspergillus niger*, *Aspergillus welwitschiae*, *Aspergillus steynii*, *Aspergillus westerdijkiae*, and *Penicillium verrucosum* and AF and OTA production under different temperature regiments. Data were treated by ANOVA, and machine learning (ML) models able to predict the growth inhibition percentage were built, and their performance was compared. All factors LAB strain, fungal species, and temperature significantly affected fungal growth and mycotoxin production. The fungal growth inhibition range was 0–100%. Overall, the most sensitive fungi to LAB treatments were *P. verrucosum* and *A. steynii*, while the least sensitive were *A. niger* and *A. welwitschiae*. The LAB strains with the highest antifungal activity were *Pediococcus pentosaceus* (strains S11sMM and M9MM5b). The reduction range for AF was 19.0% (aflatoxin B1)-60.8% (aflatoxin B2) and for OTA, 7.3–100%, depending on the bacterial and fungal strains and temperatures. The LAB strains with the highest anti-AF activity were the three strains of *P. pentosaceus* and *Leuconostoc mesenteroides* ssp. *dextranicum* (T2MM3), and those with the highest anti-OTA activity were *Leuconostoc paracasei* ssp. *paracasei* (3T3R1) and *L. mesenteroides* ssp. *dextranicum* (T2MM3). The best ML methods in predicting fungal growth inhibition were multilayer perceptron neural networks, followed by random forest. Due to anti-fungal and anti-mycotoxin capacity, the LABs strains used in this study could be good candidates as biocontrol agents against aflatoxigenic and ochratoxigenic fungi and AFL and OTA accumulation.

## 1. Introduction

Mycotoxins are secondary metabolites of filamentous fungi that can cause diseases in humans and animals. Although filamentous fungi can collectively produce hundreds of mycotoxins, in the EU, only a few of them are subject to regulation [1]. In terms of acute and chronic toxicity for humans and animals, the most relevant mycotoxins are aflatoxins (AF). AF are produced by various species of *Aspergillus* subgenus *Circumdati* section *Flavi*, but the two species of greatest concern are *Aspergillus flavus* and *Aspergillus parasiticus* [2]. *A. flavus* produces aflatoxin B1 (AFB1) and B2 (AFB2), and *A. parasiticus* can produce AFB1, AFB2, aflatoxins G1 (AFG1), and G2 (AFG2), although some reports indicate that *A. flavus* strains can also synthesize the G-type AF [3]. AF are present in very important food with many foods, such as cereals and nuts [4,5,6], breakfast cereals [7], infant foods [8], cocoa [9], legumes [10], or milk [11], among others. For a long time, AFB1 was listed by the International Agency for Research on Cancer (IARC) [12] as carcinogenic to humans. In addition to hepatocellular carcinoma, AF are associated with occasional outbreaks of acute aflatoxicosis, which lead to death shortly after exposure. Approximately 4500 million people living in developing countries are chronically exposed to AF through contaminated diets. In Kenya, acute AF intoxication leads to liver failure and, ultimately, death in around 40% of the cases [13]. Thus, the presence of these fungi and AF in food and feed is an important concern for manufacturers, consumers, researchers, and regulatory agencies.

After AF, ochratoxins are the most relevant mycotoxins produced by *Aspergillus* spp., particularly ochratoxin A (OTA). This mycotoxin is produced mainly by *Aspergillus steynii* and *Aspergillus westerdijkiae* [14], *Aspergillus niger, Aspergillus welwitschiae,* and *Aspergillus carbonarius* [15,16], and *Penicillium* species, mainly *Penicillium verrucosum* [17,18]. OTA is frequently found in cereals [19,20,21,22], beer and wine [23], grapes [24], cheeses [25], cured meat products [26], bread [27], coffee [28], cocoa [29], dried fruits [30], and spices [31]. OTA acts as a potent nephrotoxin [32] and also exhibits teratogenic, embryotoxic, genotoxic, neurotoxic, and immunosuppressive effects [33,34]. It has been classified as a possible human carcinogen by the IARC [12].

*A. flavus* and *A. niger* are reported, after *Aspergillus fumigatus,* as the second leading cause of invasive aspergillosis and the most common cause of superficial infection. It is estimated that 1.5 to 2 million people die of a fungal infection each year, many of which are caused by these *Aspergillus* spp. [35,36,37].

Some European countries are major producers and exporters of cereals, but their economies and trade have been severely affected because of contaminated products by mycotoxins in recent decades. According to the Food and Agriculture Organization, 25% of the world’s food crops are badly affected by mycotoxins either during growth or storage [38,39]. Moreover, globally, fungal infections of the most cultivated food crops were assessed to annihilate about 125 million tons of products annually [40]. Fungal attacks on cereals entail an annual loss of about $60 billion in global agricultural production [41]. In this context, the design of strategies capable of controlling the growth of toxigenic fungi and minimizing the presence of mycotoxins in food, particularly in cereals, is an urgent need. Though different physical and chemical methods are used [42,43,44], the data on economic losses, illnesses, and death in the world warn that the risks associated with contamination by fungi and mycotoxins in food remain unsolved.

Temperature is a key environmental factor that influences both the rate of fungal spoilage and the production of mycotoxins. Based on the predictive model developed for *A. flavus* growth and AFB1 production linked to crop phenology data, the risk of AF contamination was assessed to have a chance of increasing in maize in the future, due to the climate change trend. In the +2 °C climate change scenario, there is a clear increase in AF risk in areas such as central and southern Spain, southern Italy, Greece, northern and southeastern Portugal, Bulgaria, Albania, Cyprus, and European Turkey, as compared to the actual current temperature [45,46].

It has been reported that, in the last few years, mycotoxin levels in cereals have increased in Europe, which is probably due to the adaptive abilities of the toxigenic fungi, especially *A. flavus*, to the higher temperatures associated with climate change [45].

Lactic acid bacteria (LAB) have been broadly used in conventional food fermentations since ancient times and are considered GRAS, and many of them are classified as Qualified Presumption of Safety by the American Food and Drug Agency and the European Food Safety Authority, respectively [47]. Numerous LAB are considered “Green preservatives”, due to their potential to retard fungal contamination in food [48]. Currently, consumers have an increasing demand for ‘natural’ and ‘healthy’ foods, and LAB are a promising alternative for the biocontrol of toxigenic fungi [48]. The role of LAB is not limited to inhibiting fungal growth, but some LAB strains can interact with mycotoxins, causing their inactivation or removal [48,49,50].

Few reports have shown the antifungal effect of selected LAB strains against aflatoxigenic or ochratoxigenic fungi; only in a few of them, the effect of LAB on the production of AF or OTA is studied, and in none of them, the effect of temperature on the growth and mycotoxin production in dual culture bacteria-fungus has been investigated; in addition, predictive models have not been applied to these systems [48,49].

Machine learning (ML) is a subfield under artificial intelligence that constitutes a challenge and a great opportunity in numerous scientific, technical, and clinical disciplines. ML investigates algorithms able to learn autonomously, directly from the data. ML refers to the process of fitting predictive models to data or identifying informative clusters within data. The field of ML essentially attempts to approximate or mimic the human ability to recognize patterns using computation [51]. ML methods can be supervised and unsupervised. Supervised ML concerns the fitting of a model to data that have been labeled, where there exists some ground truth property, which is experimentally measured [51]. Supervised learning can be grouped into regression and classification. The dataset where ML works is composed of many data points, each of which consists of an experimental observation. For regression tasks, in the dataset, there are continuous or categorical input variables and output variables associated with the former, which are the focus of the researcher’s interest (targets). Most of the dataset (inputs and the related outputs) is used to train the ML, another part is used for model validation, and another part (the test set) is used to evaluate the built model’s performance, with data unseen during training. K-fold cross-validation is usually performed, with K being the number of groups in which the training set is divided to train/validate the model. ML algorithms iteratively make predictions of the output on the training data to establish optimum algorithm parameters. The calculated output values differ from the experimental values, and a loss function computes the average difference between them. Upon changing the values of their parameters, the algorithm tries to minimize that difference, but the process must avoid the problem of overfitting the data, which may prevent the generalization from making predictions ahead of the data used. The process ends when the loss function reaches a satisfactory average minimum difference between the predicted and experimental values. The number of applications of ML methods in most research areas is rapidly increasing. ML has been applied to every microbiology research [51,52]. In the field of predictive mycology, the most applied ML algorithms are artificial neural networks (NN), especially multilayer perceptrons (MLP), random forest (RF), support vector machines (SVM), or gradient boosting algorithms, such as extreme gradient boosting trees (XGBoost).

The objectives of the present study were (i) to evaluate the ability of eleven previously selected LAB strains, to inhibit the development of eight relevant species of aflatoxigenic or ochratoxigenic fungi in vitro under different temperature regimes, (ii) to assess the ability of the LAB strains to reduce AF and OTA levels in the media at the assayed temperatures, and (iii) to test the ability of models developed using various ML algorithms to predict the efficacy of LAB strains to inhibit or reduce the growth of the assayed fungi.

## 2. Results

### 2.1. Effect of LAB on Fungal Growth in Dual Medium MRS-CYA20S

In the controls, fungal growth became visible after a lag phase ranging from 1.5 to 3 days of incubation, depending on the temperature and fungal species.

In treatments, all LAB strains partially inhibited fungal growth. The measurements of the inhibition halos of fungal growth (transverse and longitudinal) were the same at 5 and 10 days of incubation. In the absence of LAB (controls), all fungal isolates grew and filled the Petri dishes within the incubation period at any of the three temperatures tested (20, 25, and 30 °C). Thus, the growth inhibition percentage (GIP) in the controls was zero.

**Figure 1:**
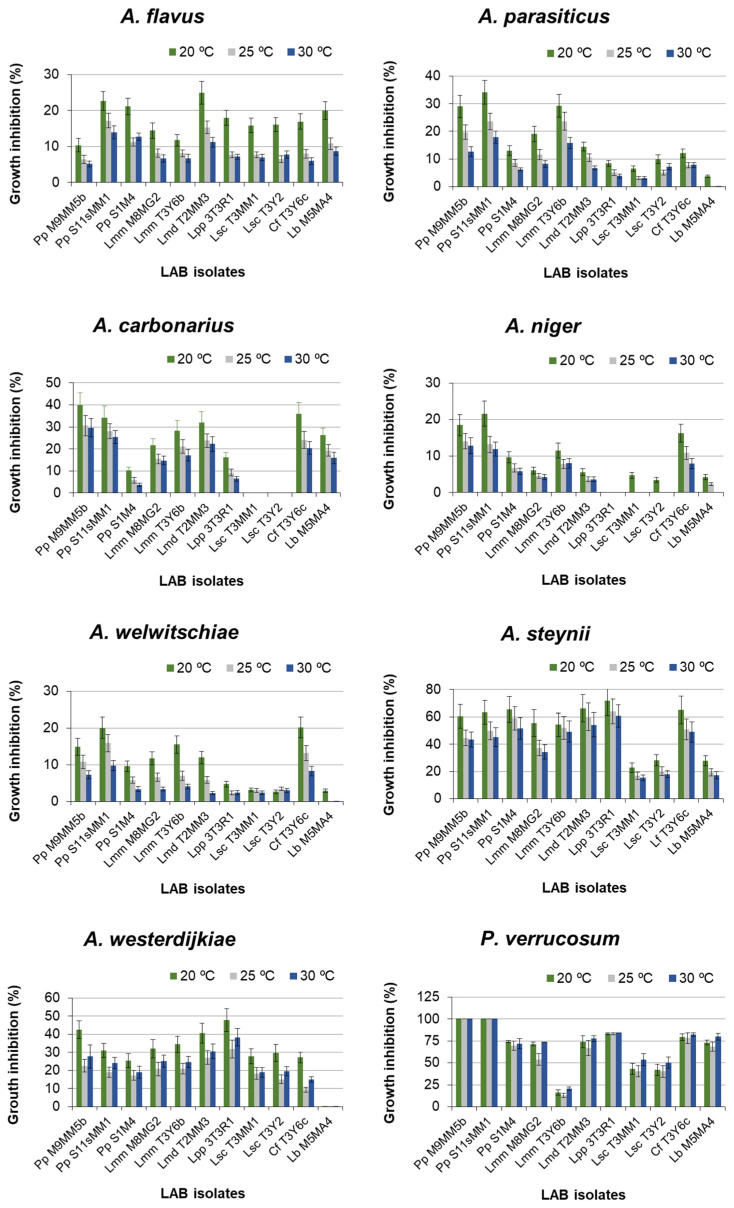
Growth inhibition percentages (GIP) of *Aspergillus* spp. and *P. verrucosum* in dual medium MRS agar-Czapek Yeast 20% Sucrose (MRS-CY20S) agar in the presence of LAB strains and cultured at 20, 25, and 30 °C. Error bars represent standard deviations. Incubation time: 10 days. For abbreviations, see the Abbreviation list of the LAB species. Pp, *Pediococcus pentosaceus*; Lmm, *Leuconostoc mesenteroides* ssp. *mesenteroides*; Lmd, *Leuconostoc mesenteroides* ssp. *dextranicum*; Lpp, *Lacticaseibacillus paracasei* ssp. *paracasei*; Lsc, *Latilactobacillus sakei* ssp. *carnosus*; Cf, *Companilactobacillus farciminis*; Lb, *Levilactobacillus brevis*. The number after the abbreviation is the strain number.

Figure 1 shows the results of the GIP, relative to the pertinent controls for *A. flavus, A. parasiticus, A. carbonarius, A. niger, A. welwitschiae, A. steynii, A. westerdijkiae,* and *P. verrucosum* in dual medium (MRS agar-Czapek Yeast 20% Sucrose (CY20S) agar) in treatments with LAB strains. The values of these GIP depended on the incubation temperature, so that, for *Aspergillus* spp., the usual order was 20 °C > 25 °C > 30 °C. However, in the cultures of *A. westerdijkiae,* the GIP was higher at 30 °C than at 25 °C. In the cultures of *P. verrucosum,* there was not a large influence of the incubation temperature on inhibition data, although on average, the fungus grew better at 25 °C and worse at 30 °C. In other fungi with some LAB, the GIP at 30 °C was a bit higher than at 25 °C. Regardless of temperature and LAB strains, the GIP ranges were 5–25% for *A. flavus*, 0–35% for *A. parasiticus*, 0–40% for *A. carbonarius*, 0–22% for *A. niger*, 2–20% for *A. welwitschiae*, 15–70% for *A. steynii*, 9–47% for *A. westerdijkiae*, and 10–100% for *P. verrucosum* (Figure 1).

The inhibition data were treated by multifactor ANOVA, which revealed that all the main factors (fungal species, incubation temperature, and LAB strain) significantly influenced the GIP (*p* < 0.001). First- and second-order interactions between these factors were also significant (*p* < 0.05). The influence of the fungal species on the GIP is manifested by the arrangement of the assayed fungi in six homogeneous groups, according to *post-hoc* Duncan’s test (Table 1).

In general, the most susceptible fungal species to the LAB strains was *P. verrucosum* and, in second place, *A. steynii*. The least susceptible species were *A. niger* and *A. welwitschiae* (they did not differ significantly from each other). *A. flavus* and *A. parasiticus* were scarcely susceptible, with no significant difference between them.

The influence of the bacterial strain on the growth of the studied fungi is shown in Figure 1. Only *P. verrucosum* growth was totally inhibited by two strains of *P. pentosaceus*. There were statistical differences among the LAB strains (*p* < 0.001). *Post-hoc* Duncan’s test arranged these bacteria into eight homogeneous groups (Table 2).

In general, considering all the tested fungi, the LAB strain with the highest efficacy in inhibiting the growth of the tested fungal species was *P. pentosaceus* (S11sMM1), followed by *P. pentosaceus* (M9MM5b), and then followed by *L. mesenteroides* ssp. *dextranicum* and *C. farciminis*. Conversely, the least effective LAB strains were the two strains of *L. sakei* ssp. *carnosus*, followed by *L. brevis* (Table 2). The efficacy of each LAB strain against each of the tested fungi was studied by ANOVA.

For every tested fungal species, both the factors LAB strain and incubation temperature significantly influenced the GIP. Therefore, the efficacy of each LAB depended on the fungal species and the incubation temperature. The results of *post-hoc* Duncan’s analysis are shown in Table 3. The LAB strain that showed the highest efficacy against one fungal species was not always the most effective against the other species.

Temperature also significantly influenced the growth inhibition effectiveness of LAB strains on the tested fungi. Overall, the highest effectiveness of LAB strains occurred at 20 °C, with a mean GIP close to 29%, while no significant difference was observed between 25 and 30 °C.

### 2.2. Machine Learning Approach to Model Fungal Growth Inhibition

Different ML algorithms were applied to the dataset made of input variables (fungal species, LAB strains, and incubation temperatures) and the correspondent output variable log_10_ (x + 1), where x is the mean GIP, in order to explore the ability of such algorithms to build models able to predict the GIP as accurately as possible. The categorical variables were converted into numerical “dummy” variables before computation. The multiple linear regression (MLR), MLP, RF, and XGBoost algorithms were comparatively tested.

The training was performed by 10-fold cross-validation, using 75% of the samples randomly taken from the whole dataset. The criterion for selecting the best model was based on minimizing the chosen loss function, which was the minimum root mean square error (RMSE) value for a test set that is not used to create the model during the training/cross-validation step. The R^2^ value obtained in the predictive task on the test set was also considered because a high value (near 1) indicates that the predictor variables in the regression model can well-predict the variance in the output variable.

Table 4 lists the results of the best models designed by the algorithms applied for predicting the GIP on the same test set made of 72 blind samples (25% of the dataset). An MLP with a single layer of seven hidden nodes (neurons) and *decay* of 0.01 provided the best model, with an RMSE of 0.1999 and an R^2^ value of 0.9232. An RF algorithm with *mtry* = 3 (the maximum number of selected predictor variables) and *ntry* = 500 gave the second better model, in order of performance. The XGBoost proved the worst algorithm, in spite of its large number of parameters to be tuned. RMSE and R^2^ were calculated on the basis of the log-transformed values of the GIP.

### 2.3. Effect of the LAB Strains on Mycotoxin Production

#### 2.3.1. Results of the Validation of the Method for Mycotoxin Determination

Table 5 shows the retention times and the limits of detection and quantification of the chromatographic method followed. The mean values of the recovery at three different levels for each analyte and the relative standard deviation of the recovery at these levels are also given.

#### 2.3.2. Mycotoxin Production in Controls and Cultures Containing LAB

1. Aflatoxins

**Figure 2:**
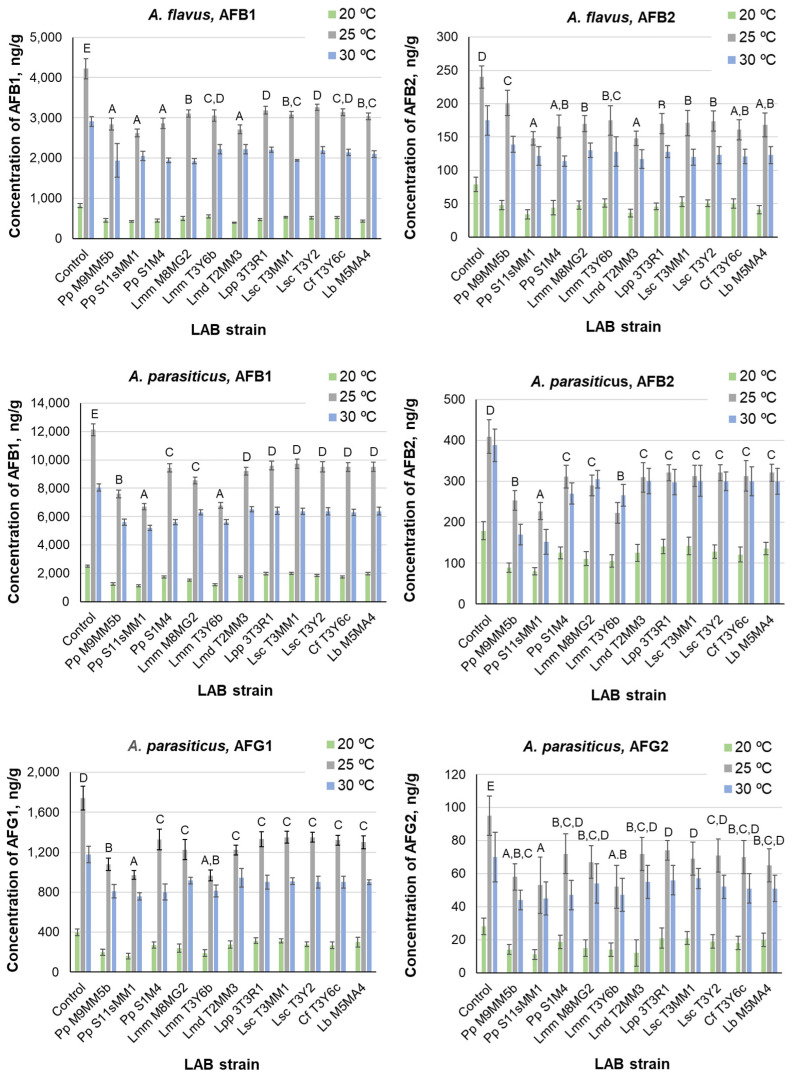
Mean concentrations of aflatoxins produced by *A. flavus* and *A. parasiticus* in dual medium MRS-CY20S agar in the presence of LAB strains and cultured at 20, 25, and 30 °C. Error bars represent standard deviations. Incubation time: 10 days. Capital letters above the bars indicate Duncan’s homogeneous groups of LAB strains. The presence of more than one letter separated by commas means that a LAB strain may be included in more than one group. For LAB abbreviations, see the legend of Figure 1 and the Abbreviation list of the LAB species.

The mean concentrations and standard deviations of AFB1 and AFB2 for the *A. flavus* and *A. parasiticus* isolates, as well as those of AFG1 and AFG2 for the *A. parasiticus* isolate (*A. flavus* did not produce AFG1 or AFG2), were determined by UPLC-MS/MS in both controls and treatments, with LAB strains on the last day of incubation. The results appear in Figure 2. The levels of these mycotoxins were lower in cultures in which LAB strains were present than in the controls without LAB strains incubated at the same temperature. *A. parasiticus* produced higher AFB1 and AFB2 concentrations than *A. flavus* at the same temperatures. The maximum average AFB1 production by *A. flavus* was 4220 ng/g in the control culture grown at 25 °C, whereas *A. parasiticus* produced 12,120 ng/g under the same conditions. The highest levels of AFB2 in the *A. flavus* and *A. parasiticus* control cultures were also recorded at 25 °C and were 240 and 409 ng/g, respectively. ANOVA indicated that both species differed significantly in AFB1 and AFB2 production.

In treatments, the factors LAB strain and temperature showed a significant influence on AF level in *A. flavus* and *A. parasiticus* (*p* < 0.01). The interaction between these two factors was also significant. Concerning AFB1 production in the treatment cultures of both species, the LAB strains were arranged into four homogeneous groups by Duncan’s test (Figure 2, denoted by letters A to D). Considering AFB2, there were three homogeneous groups of LAB (Figure 2, denoted by letters A to C). The cluster of LAB strains giving rise to lower AFB1 production in *A. flavus* (Figure 2, group A) was constituted by the three strains of *P. pentosaceus* and *L. mesenteroides* ssp. *dextranicum* (T2MM3). Therefore, these strains were the most effective for reducing AFB1 production.

The AFG1 level exceeded the AFG2 level at all the temperatures, even in the controls, where the highest AFG1 concentration was 1740 ng/g at 25 °C, whereas the AFG2 concentration was 95 ng/g, also at 25 °C. In treatments, the AFG1 levels were always below 1560 ng/g, and those of AFG2 were below 75 ng/g. The LAB strains were arranged in three and four groups, respectively, by Duncan’s test. In the case of AFG2, there was a high degree of overlapping (Figure 2).

*P. pentosaceus* (S11sMM1 and M9MM5b) and *L. mesenteroides* ssp. *mesenteroides* (T3Y6B) hindered AFG1 and AFG2 accumulation more than the remaining strains. The temperature significantly influenced the production of AF by the two fungi assayed, and the order of AF production was 25 °C > 30 °C > 20 °C, which is contrary to mycelium development. Table 6 lists the minimum and maximum percentages of AF reduction, compared to the controls in the LAB treatments of *A. flavus* and *A. parasiticus*. They depended on the incubation temperature and the fungal and LAB species. The percentage of mycotoxin reduction was calculated as 100 × [(mycotoxin level in control − mycotoxin level in treatment)/mycotoxin level in control].

For AFB1, the lowest reduction took place in *A. parasiticus* cultures at 30 °C (19.0%), and the maximum reduction occurred in *A. parasiticus* cultures at 20 °C (55.0%). For AFB2, the minimum reduction was observed in *A. flavus* cultures at 25 °C (16.2%), and the maximum reduction occurred in *A. parasiticus* cultures at 30 °C (60.8%). For AFG1 and AFG2, the minimum and maximum reductions occurred at 30 °C and 20 °C, respectively.

2. Ochratoxin A

**Figure 3:**
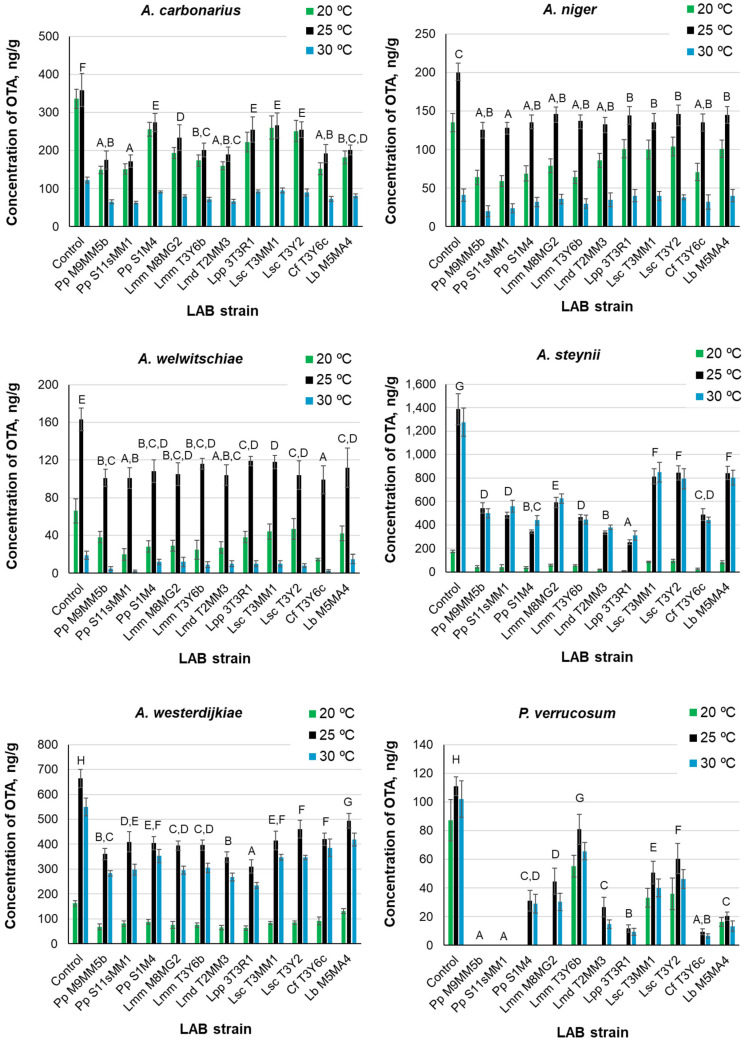
Concentrations of ochratoxin A (OTA) produced by various ochratoxigenic *Aspergillus* spp. and *P. verrucosum* in dual medium MRS agar-Czapek Yeast 20% Sucrose agar in the presence of LAB strains and cultured at 20, 25, and 30 °C. Error bars represent standard deviations. Incubation time: 10 days. Capital letters above the bars indicate Duncan’s homogeneous groups of LAB strains. The presence of more than one letter separated by commas means that a LAB strain may be included in more than one group. For LAB abbreviations, see the legend of Figure 1 and the Abbreviation list of the LAB species.

The production of OTA in control and treatment cultures at the three tested temperatures by *A. carbonarius, A. niger, A. welwitschiae, A. steynii, A. westerdijkiae*, and *P. verrucosum* are shown in Figure 3. The OTA levels were always higher in the controls than in the treatments under the same temperatures. The OTA level was the maximum in the control cultures of *A. steynii*, where the highest concentration (near 1400 ng/g) was attained at 25 °C. The following fungus, in order of OTA level, was *A. westerdijkiae* (about 670 ng/g at 25 °C in the control culture). *P. verrucosum* provided the lowest OTA concentrations.

ANOVA revealed that the three factors (fungal species, LAB strain, and temperature) significantly influenced the OTA level in the cultures (*p* < 0.01). The mutual interactions between these factors were also significant. If we consider the whole dataset, the overall order of OTA production varied with temperature as follows: 25 °C > 30 °C > 20 °C. However, the order of OTA production with temperature was different, depending on the fungal species assayed. For the black aspergilli (*A. carbonarius, A. niger,* and *A. welwitschiae)*, it was: 25 °C > 20 °C > 30 °C, but for *A. steynii*, *A. westerdijkiae*, and *P. verrucosum*, the order was: 25 °C > 30 °C > 20 °C. There were significant differences among the fungal species. Table 7 shows the arrangement of fungi in homogeneous groups obtained by Duncan’s test.

Considering the mean OTA levels in all treatment cultures of ochratoxigenic fungi at the three temperatures, the LAB strains were clustered in four homogeneous groups by Duncan’s test. They were in the order of increasing OTA levels, as follows: group A: *L. paracasei* ssp. *paracasei* (3T3R1) and *L. mesenteroides* ssp. *dextranicum* (T2MM3); group B: *P. pentosaceus* (S11sMM1 and M9MM5b), *C. farciminis* (T3Y6c), *P. pentosaceus* (S1M4), and *L. mesenteroides* ssp. *mesenteroides* (T3Y6b); group C: *L. mesenteroides* ssp. *mesenteroides* (M8MG2), and group D: *L. brevis* (M5MA4) and *L. sakei* ssp. *carnosus* (T3MM1 and T3Y2).

Each fungus was placed in a single group (A to F) without overlapping. The arrangement of LAB strains into homogeneous groups, denoted by capital letters, can be observed in Figure 3. All strains in group A hampered OTA production more than those placed in the remaining groups. *P. pentosaceus* (S11sMM1) belonged to group A in cultures of *A. carbonarius*, *A. niger*, *A. welwitschiae*, and *P. verrucosum;* however, this strain was included in group D in cultures of *A. steynii* and *A. westerdijkiae. P. pentosaceus* (M9MM5b) behaved similarly. On the contrary, *L. paracasei* ssp. *paracasei* (3t3R1) belonged to group E in cultures of *A. carbonarius*, to group C in cultures of *A. welwitschiae* and *P. verrucosum,* and to group B in cultures of *A. niger*, but it was included in group A in cultures of *A. steynii* and *A. westerdijkiae.* Table 8 lists the minimum and maximum percentages of OTA reduction in the LAB treatments of the ochratoxigenic fungi, compared to the controls. These values changed with temperature and were dependent on the LAB strains. The minimum reduction was attained in *A. niger* cultures at 30 °C and occurred in treatments with the two strains of *L. sakei* ssp. *carnosus*, *L. brevis* (M5MA4), and *L. paracasei* ssp. *paracasei* (3T3R1). This agrees with the low ability of these LAB strains to hinder *A. niger* growth (Figure 1 and Table 3). The maximum reduction (100%) was reached in *P. verrucosum* cultures, with *P. pentosaceus* (M9MM5b and S11sMM1) at all the assayed temperatures, which also agrees with their high efficacy to hinder the growth of this fungus (Table 3).

## 3. Discussion

Research approaches for bio-control agents have mainly focused on LAB, due to their antifungal effects and their GRAS status. However, in toxigenic fungi research, it is necessary to also study the effect of bacteria on mycotoxin production, since LAB modulate the growth environment of fungi, which may affect growth and mycotoxin production differently [53]. Likewise, environmental factors, mainly temperature, can play a fundamental role in these possible interactions that are established in mixed bacteria-fungi cultures. In the present study, eleven strains of different LAB species previously selected for their antifungal activity were tested, and their effects on the biocontrol of relevant aflatoxigenic or ochratoxigenic fungal species and the production of AF and OTA under different temperature regimes were studied. The assays were performed in dual-layer cultures composed of MRS agar (to promote bacterial growth) and CYA20S (to promote fungal growth and mycotoxin production).

It is known that the characteristics of the medium/food and microorganisms (both fungi and LAB strains) contribute to the detoxification properties of LAB [48,49]. However, a comparative study of our results with those found in the bibliography is difficult because of the different species/microbial strains and environmental conditions. Moreover, some studies analyzed the effects of LAB strains on the growth of aflatoxigenic or ochratoxigenic fungi or on the production of one or more AF or OTA, but not on both processes at the same time, and the interactive effect of environmental temperature was not studied either [48,49]. Therefore, we will try to discuss and compare the results obtained in the present study with those previously reported for other strains/species of LAB and aflatoxigenic or ochratoxigenic fungi, regardless of the experimental conditions employed.

Some authors have reported the antifungal activity of the selected LAB strains against aflatoxigenic or ochratoxigenic fungi; for example, *Lactobacillus fermentum* (YML014) isolated from Nigerian fermented foods was active against *A. flavus* and *A. niger* (reduction of fungal mycelia by ~50% in liquid medium) [54]. The mycelial dry weight of *A. flavus* was reduced to 73 and 85% using *L. casei* CRL 431 (isolated from human feces) and *L. rhamnosus* CRL 1224 (isolated from yogurt), respectively [55]. *Lactobacillus brevis* (LPBB03) isolated from coffee fruits inhibited the growth of *A. westerdijkiae* (over 50%) [56]. Other studies have shown that fermentation products of LAB can reduce the growth of aflatoxigenic or ochratoxigenic fungi. In this way, the fermentation products of *Leuconostoc citreum*, *Lactobacillus rossiae*, and *Weissella cibaria,* isolated from Italian durum wheat semolina, with high content in lactic acid and acetic acid, inhibited the growth of *A. niger*, *Penicillium roqueforti*, and *Endomyces fibuliger*, near 100% [57]. Bread spoilage caused by *A. niger* was remarkably decreased using a strain of *Lactobacillus reuteri* isolated from whole wheat sourdough. The bioactive ingredients produced by the bacterium were identified as n-decanoic acid, 3-hydroxydecanoic acid, and 3-hydroxydodecanoic acid [58]. The use of phenyl lactic acid (PLA) produced by *Pediococcus acidilactici* (CRL 1753), together with calcium propionate (CP), in bread-making as a bio-preserver significantly increased the shelf life of bread. At 18 days of storage, no molds were observed, while 70% of pieces of bread treated with CP alone were spoiled by *A. niger* and other fungi [59]. In our study, the results show that all assayed LAB strains reduced the growth of *A. flavus* (Af2225), *A. parasiticus* (Ap02), *A. carbonarius* (Ac12g), *A. niger* (An07g), *A. welwitschiae* (Aw11g), *A. steynii* (As1w), *A. westerdijkiae* (Aw019), and *P. verrucosum* (Pv10w) in percentages ranging from 0 (*A. steynii*) to 100% (*P. verrucosum*). Overall, the LAB strain encompassing the most antifungal activity was *P. pentosaceus* (S11sMM1), followed by *P. pentosaceus* (M9MM5b) and, in the third place, *L. mesenteroides* ssp. *dextranicum* (T2MM3) and *C. farciminis* (T3Y6c). The least effective LAB strains were *L. sakei* ssp. *carnosus* (T3MM1 and T3Y2). Additionally, the effectivity of each LAB strain may be quite different, depending on the fungal isolate. The fungal isolates most resistant to LAB treatments were *A. niger* and *A. welwitschiae*, followed by *A. flavus* and *A. parasiticus.* Conversely, *P. verrucosum* was the most sensitive fungus, while the remaining isolates showed intermediate susceptibility.

Concerning the use of ML to develop models able to predict, as accurately as possible, the output values (GIP) as a function of the input variables, it has been found that an MLP (NN) gave the best results because of the lowest RMSE (0.1999) and the highest R^2^ value (0.9232) for the test set. After the detransformation of the logarithmic approach, the RMSE was 0.585%. It was followed by RF, then by MLR, and the XGBoost, which proved to be the worst algorithm, in spite of its major complexity. There are few reports concerning ML applications in the field of predictive mycology. Models designed by RF were superior to those built by MLP, MLR, and XGBoost to predict the growth rate (GR) of *Fusarium culmorum* and *F. proliferatum* cultured on partly milled maize with ethylene-vinyl alcohol copolymer (EVOH) films containing pure active components of essential oils [60]. However, XGBoost was better than other assayed algorithms (RF, MLR, MLP, SVM) for predicting the GR of *F. sporotrichioides* in oat grain under the influence of EVOH films containing pure active components of essential oils [61]. XGBoost also performed better than other algorithms (RF, MLR, MLP) for predicting the GR of *F. culmorum* and *F. proliferatum* in cultures carried out in a maize extract medium treated with antifungal formulations at two temperatures and two water activities [62]. These differences may be due to differences in the structure of the datasets. Thus, it is difficult to ensure a priori which ML algorithm will have the average minimum error in predicting the GR or the GIP of the fungi. It is necessary to compare them using the same dataset. There are not many reports concerning the application of ML to predict the growth of fungi in food or food-related substrates. Wawrzyniak [63] found that the MLP can be applied to develop a model able to predict the fungal population levels in bulk-stored rapeseeds at various temperatures (12–30 °C) and water activity (0.75–0.90) with high levels of generalization capability and prediction accuracy. This agrees with the study of Panagou and Kodogiannis [64] for predicting the maximum specific GR of *Monascus ruber*. However, the assessment of ML models for predicting the growth of *Aspergillus* spp. or *P. verrucosum* in the presence of LAB strains has been carried out for the first time in the present study.

These and other research, among which the present study is an important contribution, demonstrate that both the selected LAB strains and their metabolism products can be extraordinarily useful in the biocontrol of aflatoxigenic and ochratoxigenic species.

In our study, all the tested LAB strains produced a reduction of AF levels, compared to the controls. For each fungus, the reduction rate was dependent on the temperature and LAB strain. For AFB1, the ranges of reduction of the concentration in the medium were 19.0–55.0% for *A. parasiticus* and 22.8–52.3% for *A. flavus*. For AFB2, the ranges of reduction of the concentration in the medium were 20.7–60.8% for *A. parasiticus* and 16.2–57.0% for *A. flavus*. The most efficient LAB strains in reducing AF levels in the medium were *P. pentosaceus* (S11sMM1) for AFB1 in cultures of *A. parasiticus* and for AFB2 in cultures of both species and *L. mesenteroides* ssp. *dextranicum* (T2MM3) for AFB1 in cultures of *A. flavus*. This agrees with the effectiveness of these strains, especially the former, for reducing the growth of *A. flavus* and *A. parasiticus*. The reduction of OTA levels ranged from 7.3% for *A. niger* to 100% for *P. verrucosum*. The most efficient LAB strains for reducing OTA in the medium were *L. paracasei* ssp. *paracasei* (3T3R1) and *L. mesenteroides* ssp. *dextranicum* (T2MM3), whereas the least efficient were the two strains of *L. sakei* ssp. *carnosus* and *L. brevis* (M5MA4).

Some previous reports focused on the study of the effect of the selected LAB strains on the inhibition or elimination of AF or OTA in the media. Thus, *L. casei* (L30) bound up to 49.2% of the available AFB1 in aqueous solution [65]. *L. casei* Shirota (mainly live bacteria) bound AFB1 to their cell components with an efficacy of up to 90% [66]. *Lactococcus lactis* ssp. *cremoris*, *Lactobacillus rhamnosus*, and *L. lactis* ssp. *lactis* exhibited high binding capacities to remove AFM1 in milk at levels of 81.4, 56.8, and 50.8%, respectively [67], and selected isolates of *L. fermentum* (LC3/a, LC4/c, LC/5a, and LM13/b), from curd samples, were effective in removing AFB1 from culture media above 75% [68]. Some assays have shown the elimination of AF during fermentation. *L. kefiri* (KFLM3), isolated from kefir grains, was able to adsorb 80–100% AFB1, zearalenone, and OTA when cultivated in milk [69]. *L. plantarum* (R2014 and EQ12), *L. buchneri* (R1102), and *P. acidilactici* (R2142 and EQ01) linearly decreased the initial concentration of AFB1 (30 µg/kg) in silage corn forage after 3 days to <0.35 µg/kg [70].

Regarding the effect of LAB on OTA production by ochratoxigenic fungi, or their elimination from the medium, the present work provides relevant information that complements the previous studies and broadens the spectrum of the possible LAB species/strains that are useful in the food industry. In a liquid medium, *Lactobacillus acidophilus* (VM 20) produced a decrease of OTA level ≥ 95% by adsorption of the toxin [71]. Different *Oenococcus oeni* strains removed OTA from the medium at levels > 60%, with significant differences depending on the strain, incubation period, initial OTA level, and pH. Toxin removal was independent of bacterial viability [72,73]. *L. plantarum*, *L. brevis*, and *L. sanfranciscensis* reduced the OTA level from 16.9% to 35% in the MRS medium after 24 h of contact. OTA binding was even higher in the case of thermally inactivated bacterial biomass [74]. A high percentage of OTA reduction by *L. plantarum* (LabN10), *L. graminis* (LabN11) (>97%), and *P. pentosaceus* (>81.5%) was recorded. Factors such as temperature, pH, and bacterial biomass showed a significant effect on the growth of *A. carbonarius* (ANC89) and OTA levels in the medium [75]. These studies show the high efficacy of LAB in the control of AF and/or OTA in culture media and in food, as well as their high ability as detoxification agents.

Furthermore, no stimulation of AF or OTA production was detected in any treatment with the LAB strains used in the present work, with respect to the controls. On the contrary, in all treatments, a positive relationship between the GIP and the reduction of AF or OTA levels in the cultures was observed (Figure 1 and Table 6 and Table 8). Moreover, the percentage of mycotoxin reduction was always higher than the GIP under the same conditions. It suggests the diffusion in the solid medium of possible antifungal and, especially, “antimycotoxin” metabolites, such as lactic acid, benzoic acid, propionic acid, formic acid, butyric acid, hexanoic, caproic acids, phenyl lactic acid, H_2_O_2_, monohydroxy octadecenoic acid, CO_2_, cyclic dipeptides, phenolics, bacteriocins, fungicins, reuterine, ethanol, diacetyl, hydroxyl fatty acids, etc. [76,77]. Our results agree with the previous reports, where the efficacy of selected strains of other LAB species on the control of the growth of aflatoxigenic or ochratoxigenic fungi and the production of AF or OTA were analyzed at the same time. Taheur et al. [78], using the strain of *L. kefiri* (FR7), found levels of growth reduction of *A. flavus* and *A. carbonarius* of 51.67% and 45.56%, respectively. However, *L. kefiri* (FR7) more deeply impacted mycotoxin suppression, with reduction percentages reaching 97.22%, 95.27%, and 75.26% for AFB1, AFB2, and OTA, respectively. These results suggest that, in addition to the space competitivity fungi-LAB, the liberation of bacterial metabolites can contribute to the inhibition of mycotoxin production. Although in the present work, natural media are not used, the previous results point out that reduction in mycotoxin production also happens in natural matrices. Thus, inoculation of *L. kefiri* (FR7) in almonds artificially contaminated with *A. flavus* decreased 85.27% of AFB1 and 83.94% of AFB2 content after 7 incubation days. Application of *L. kefiri* (FR7) in peanuts artificially contaminated with *A. carbonarius* reduced OTA content to 25%. Similar results were obtained by Oliveira de Almeida-Moller et al. [79], who found that LAB strains, such as *L. brevis* (2QB422), *Levilactobacillus* spp. (2QB383), *L. brevis* (2QB446), and *Levilactobacillus* spp. (3QB398), affected the growth of *A. parasiticus*, and they were also able to reduce the production of at least three of the four tested AF by 50%. Surprisingly, despite no clear inhibition effect of the LAB strains on fungal growth, strong inhibition potential (>50%) on AF was shown by strains *L. plantarum* (3QB350), *L. plantarum* (1QB314), and *Levilactobacillus* spp. (3QB167). Ghanbari et al. [80] reported the effect of *L. plantarum* and *L. delbrueckii* ssp. *lactis* on the simultaneous reduction of the growth of *A. parasiticus* (ATCC15517), AF production (mainly AFG2), and the level of aflR gene expression. Gomaa et al. [81] found that *L. brevis* reduced the growth of *A. flavus* and AFB1 production by about 96%. The reduction effect was also confirmed at the transcriptional level, as well, where 80% less expression of the omt-A gene was observed, as compared to the control [82]. Strains of *P. pentosaceus* and *L. plantarum* isolated from grapes showed good antifungal activity against the *A. niger* aggregate and *A. carbonarius*. *P. pentosaceus* (RG7B) showed promising potential probiotic characteristics and had a high ability for OTA removal after 48 h of incubation in MRS (84%).

## 4. Conclusions

The selected LAB strains used in the present study can be excellent tools for the simultaneous biocontrol of the main aflatoxigenic and ochratoxigenic fungal species that affect cereals and grapes, as well as for the reduction/inhibition of the production of AF and OTA in the medium. The environmental temperature is a parameter with a very significant influence on these processes. In treatments with the assayed LAB strains, no stimulation of AF or OTA production, compared with the controls, was observed. Due to the anti-fungal and anti-mycotoxin capacity, the LAB strains used in this study could be good bio-preservative candidates for many food commodities. Further studies, using, in the first place, the natural matrices from which the fungal strains have been isolated (cereal grain and grapes), will be carried out in the future.

## 5. Materials and Methods

### 5.1. Reagents and Standards

Standards of mycotoxins AFB1, AFB2, AFG1, AFG2, and OTA were purchased from Sigma-Aldrich (Alcobendas, Spain). Glycerol was from Panreac Química (Castellar del Vallés, Barcelona, Spain). Potato dextrose agar (PDA) was from Scharlab (Barcelona, Spain). Agar, yeast extract, and De Man–Rogosa–Sharpe agar (MRS) were from Oxoid (Basingstoke, UK). Tween 80 was from Merck (Darmstadt, Germany). All reagents supplied were of analytical grade. Acetonitrile (ACN) and formic acid (all LC grade) were from J.T. Baker (Deventer, the Netherlands). Pure water was obtained from a Milli-Q Plus apparatus (Millipore, Billerica, MA, USA).

### 5.2. Microbial Strains and Culture Conditions

Eleven LAB strains previously selected among several hundreds of strains, in view of their antifungal activity, were assayed against relevant toxigenic and/or phytopathogenic *Aspergillus* spp. and *P. verrucosum* isolated from cereals and grapes grown in Spain. These LAB strains were: *Pediococcus pentosaceus* (M9MM5b, S11sMM1 and S1M4), *Leuconostoc mesenteroides* ssp. *mesenteroides* (M8MG2 and T3Y6b), *Leuconostoc mesenteroides* ssp. *dextranicum* (T2MM3), *Lacticaseibacillus paracasei* ssp. *paracasei* (3T3R1), *Latilactobacillus sakei* ssp. *carnosus* (T3MM1 and T3Y2), *Companilactobacillus farciminis* (T3Y6c), and *Levilactobacillus brevis* (M5MA4). All LAB strains were previously isolated from food samples in Argentina and Peru and characterized as described by Elizaquível et al. [83] and Jiménez et al. [84]. LAB strains were held at the IATA-UVEG/RA Collection (https://www.microbiospain.org/portfolio-item/iata-uveg-ra/ (accessed on 2 February 2022) and stored in MRS liquid medium containing 20% (*v/v*) glycerol at −80 °C. Before being included in this study, they were tested for authenticity by MALDI-TOF MS profiles determination using a Microflex LT MALDI-TOF MS device, software, and database for identification (Bruker Daltonics, Germany) at the Spanish-Type Culture Collection (CECT, University of Valencia, Spain). 

Nine isolates of toxigenic fungi were previously selected for their phytopathogenic and/or toxigenic capacity. They were: *A. flavus* (Af2225) and *A. parasiticus* (Ap02) (from maize), *A. carbonarius* (Ac12g), *A. niger* (An07g) and *A. welwitschiae* (Aw11g) (from grapes), *A. steynii* (As1w), and *A. westerdijkiae* (Aw019) (from wheat) and *P. verrucosum* (Pv10w). Spore suspensions of pure cultures were stored at −20 °C in glycerol/saline solution (25/75, *v/v*). Fungal isolates are held at the Mycology and Mycotoxins Group Culture Collection of Valencia University (Spain).

### 5.3. Antifungal Assays

#### 5.3.1. Inoculum of Bacterial and Fungal Preparation

Before carrying out the study, LAB isolates were grown in MRS broth at 28 °C for 3–5 days, then they were transferred to MRS agar and incubated under the same conditions. From these fresh cultures, bacterial suspensions on a saline solution (1.5 × 10^8^ CFU/mL, 0.5 McFarland turbidity standard) were prepared. Two µL of this suspension were used as inoculum in the antifungal assays.

Fungal isolates were grown on PDA at 28 °C for 7 days. From these fresh fungi cultures, a suspension of spores containing 1 × 10^6^ spores/mL was prepared in sterile pure water modified with Tween 80 (0.005%). One mL of this suspension was used as inoculum in the antifungal assays. All LAB and fungal suspensions were prepared immediately before each antifungal experiment.

#### 5.3.2. Preparation, Inoculation, and Incubation of Dual Cultures

LAB strains were assayed for antifungal activity against toxigenic fungi using a dual culture overlay assay, according to Magnusson et al. [85], with some modifications. MRS agar was prepared, autoclaved (115 °C for 30 min), and poured into Petri dishes (15.0 mL/dish). A fresh suspension of each LAB strain (as described in Section 5.3.1) was inoculated on plates containing MRS agar in two 2-cm lines (parallel and centered at ¼ and ¾ of the same diameter) (2 µL per line) and allowed to grow at 28–30 °C for 48–72 h. The plates were then overlaid with 10 mL of Czapek yeast 20% sucrose agar (CYA20S) (yeast extract 5.0 g, sucrose 200.0 g, sodium nitrate 3.0 g, dipotassium hydrogen phosphate 1.0 g, potassium chloride 0.5 g, magnesium sulfate heptahydrate 0.5 g, ferrous sulfate heptahydrate 0.01 g, agar 15.0 g, and deionized water 1000 mL). Before being poured into the plate, this medium was autoclaved at 115 °C for 30 min, allowed to cool until 45 °C, and inoculated with 1 mL of the previously prepared suspension of fungal spores (Section 5.3.1). The dual cultures (LAB plus fungus) and control cultures (without LAB) were incubated at 20, 25, and 30 °C for 5 days. After this first incubation period, the inhibition zone of fungal growth around the previously grown bacteria was measured. The inhibition halo from each LAB inoculation line was approximately elliptical, so two measurements of the halo axes (longitudinal and transverse) at right angles were taken. Then, the plates were incubated again at the same temperatures for another 5 days. At the end of the incubation period (10 days), the size of the inhibition halo was measured again. In treatments, the x and *y* axes of the two elliptical zones of fungal growth inhibition on each Petri dish were measured with a rule and averaged. The surface of the inhibition area was calculated using the formula of the area inside the ellipse. The GIP was calculated from the ratio between the area of the inhibition zone and the inner area of the dish. The upper limits for the x and *y* axes of each elliptical inhibition zone on the 90 mm diameter plate were estimated as 44 mm and 76.2 mm, respectively. When the axes reached these values, the inhibition zone was considered to be equal to the entire inner surface of the dish, and the GIP was supposed to be 100%. At the end of the incubation period, mycotoxin levels were determined in all controls and LAB treatments. Experiments were run in triplicate and repeated twice. The results were averaged.

### 5.4. Mycotoxin Determination

Before the determination of the mycotoxin concentrations in MRS agar–CYA20S cultures by ultra-high-performance liquid chromatography with detection by triple quadrupole mass spectrometry (UPLC–MS/MS), calibration and validation assays of the method were performed. The analytical method described by Romera et al. [86] was followed, with minor modifications concerning the substrate and the UPLC–MS/MS equipment.

#### 5.4.1. Calibration Solutions

Standards of AF and OTA were dissolved in ACN/water (50/50, *v/v*) to obtain stock concentrated solutions, which were maintained at −20 °C when not in use. They were appropriately diluted with ACN/water (50/50, *v/v*) to prepare the diluted standard solutions of different concentrations. Portions of non-inoculated solid control medium (MRS Agar–CYA20S) (3:2, *v/v*) were homogenized in a stomacher. Four g of the homogenate was mixed with 16 mL of ACN/water/formic acid (80:19:1, *v/v/v*) in a Falcon tube and shaken in an orbital shaker for 1 h. The mixture was centrifuged at 4260× *g* for 5 min, and aliquots of the supernatant (2.0 mL) were transferred to vials and evaporated to dryness at 40 °C under a slight stream of N_2_. To perform matrix-matched calibration, appropriate volumes of mixtures of mycotoxin standard solutions were added to the residues and then diluted (if necessary) with ACN/water/formic acid (80:19:1, *v/v/v*) up to 2.0 mL to attain the desired concentrations. Before being injected into the UPLC–MS/MS system, these standards were filtered using a syringe filter (0.22 μm, PTFE). For each mycotoxin, a calibration line was obtained by linear regression (weighting by 1/x) of the peak area from the quantifier ion vs. mycotoxin concentration. The concentration ranges of working calibration solutions (ng/mL) were as follows: AFB1 (0.57–20), AFB2 (0.57–20), AFG1 (0.57–9.25), AFG2 (0.3–9.25), and OTA (1.9–62.5).

#### 5.4.2. Mycotoxin Recovery

Method validation was carried out by analysis of blank MRS–CYA20S agar spiked with standards of the four AF and OTA (*n* = 5) at different concentrations, which was achieved by the addition of aliquots of mycotoxin standard solutions to Erlenmeyer flasks containing 10 g of autoclaved MRS–CYA20S agar (3/2 *v/v*) allowed to cool to around 45 °C. The level ranges (ng of mycotoxin/g medium) for recovery studies were 4–70 for AFB1, 4–70 for AFB2, 4–32 for AFG1, 3–32 for AFG2, and 10–100 for OTA. Once homogenized, the spiked medium was poured into Petri dishes and allowed to cool at room temperature. After solvent evaporation, the solid medium was cut into small pieces and homogenized using a stomacher. The spiked homogenate (2.0 ± 0.1 g) was extracted in a capped Falcon tube with 8 mL ACN/water/formic acid (80:19:1, *v/v/v*) in an orbital shaker for 1 h. The extracts were treated as described in 5.4.1. Concentrations were determined by interpolation of the signals in the calibration lines. Then, the mean recovery rates and the mean relative standard deviations were calculated.

#### 5.4.3. Determination of Mycotoxins in Dual Cultures MRS-CYA20S

To determine the effect of LAB on mycotoxin production, the dual cultures (substrate plus biomass of microorganisms) of 10 days in MRS agar-CYA20S agar used for antifungal assays were cut into small pieces, removed, weighed, homogenized in a stomacher, and analyzed, as described in Section 5.4.2, for spiked media to determinate the levels of AFB1 and AFB2 (*A. flavus* cultures), AFB1, AFB2, AFG1, and AFG2 (*A. parasiticus* cultures), and OTA (*A. carbonarius*, *A. niger, A. welwitschiae, A. steynii, A. westerdijkiae*, and *P. verrucosum* cultures). Mycotoxin analysis was performed as described in Section 5.4.2.

The mycotoxins (AF and OTA) accumulated in MRS–CYA20S agar by the strains of *Aspergillus* spp. and *P. verrucosum* were determined at the end of the incubation period. The whole culture in Petri dishes (substrate plus biomass of microorganisms) was cut into small pieces, removed, weighed, homogenized in a stomacher, and analyzed, as described above (Section 5.4.2), for spiked media. Extracts filtered using 0.22-μm filters were injected into the UPLC–MS/MS system. When mycotoxin levels in cultures were too high for the linear calibration range, extracts were appropriately diluted with the same solvent and injected again. Concentrations were determined by interpolation in the calibration lines obtained with standards (Section 5.4.1).

#### 5.4.4. UPLC-MS/MS Conditions

The instrument used for the separation and detection of the mycotoxins was an Exion LC AD coupled to an MS/MS Triple Quad 6500+ System, provided with an electrospray ionization source (ESI) (AB Sciex, Foster City, CA, USA) and operated in positive ion mode (ESI+). Chromatograms were obtained by multiple reaction monitoring, and the two main product ions, one quantifier (q1) and one qualifier (q3), were monitored. The injection volume was 5 µL. Separation was performed at 30 °C in an Acquity UPLC BEH C18 column (50 × 2.1 mm, 1.7 μm particle size) (waters). The mobile phase was a time-programmed gradient of solvent A (water containing 0.15 mM ammonium formate and 0.1% formic acid) and solvent B (methanol) at a constant flow rate of 0.35 mL/min. The program was 95% solvent A for 2.0 min; 0% solvent A at min 13.0 hold to min 15.0, followed by a return to the initial conditions (95% solvent A) at min 15.1 and a stabilization period up to min 18.0. The ESI and other MS/MS conditions are in Table 9. Other ESI-source parameters were as follows: source temperature: 350 °C; curtain gas: 206.843 kPa; nebulizer gas (GS1): 379.212 kPa; heating gas (GS2): 379.212 kPa; ion spray voltage: 4500 V; target cycle time: 1 s; collision gas pressure (pure nitrogen): high.

### 5.5. Statistics

Three replications were conducted for each treatment, and the treatments were repeated two times. The results were expressed as the mean value with standard error. Data were analyzed by multifactor analysis of variance (ANOVA) using Statgraphics Centurion XV.II statistical package (StatPoint, Inc., Warrenton, VA, USA). *Post-hoc* Duncan’s multiple range test (α = 0.05) was used to find homogenous groups when significant differences between means were revealed by ANOVA. For calculation purposes, undetectable mycotoxin levels were considered to be zero.

### 5.6. Method for the Design of Predictive ML Models for Growth Inhibition Percentage

Various ML models were comparatively tested using the same dataset for modeling the GIP in cultures of the different fungal species, carried out at three temperatures and treated with the different LAB strains. Control cultures were also considered. They were multilayer perceptrons (MLP), which is a kind of NN (we used a single layer perceptron), random forest (RF), extreme gradient boosting trees (XGBoost using the *xgbTree* method), and multiple linear regression (MLR). The software used was R and the ‘classification and regression training’ (caret) package [87]. The parameters to be tuned in MLP were *size* (the number of hidden nodes in the hidden layer) and *decay* (the weight decay rate set at 0.01 to 1.0); in RF, they were *ntry* (number of trees to grow, usually 500) and *mtry* (number of variables randomly sampled as candidates at each split, here 2 or 3); in XGBoost, there were more parameters, and to avoid very large computation times, some of them were kept constant, while other were tuned (Table 4). The input variables were three (temperature, LAB strain, and fungal species). The output variable was log_10_(GIP + 1). The output values were averaged, as only one output value was associated with a set of input values. Variables were preprocessed before mathematical treatment. The categorical input variables (LAB strain and fungal species) were transformed into numerical variables by recoding them as “dummy” variables [60,61].

Before training the ML models, the dataset was randomly split into training (75%) and test (25%) sets. The training set was used to develop the ML models using 10-fold cross-validation, whereas the test set was used to assess model performance using independent data hidden during the training/validation. MLR used all independent variables to predict the output value, and the coefficients were chosen by the least square method. This algorithm assumes that there is a linear relationship between the dependent and independent variables. The metric used for obtaining the best parameters and for evaluating the performance of the ML models (the loss function) was the RMSE over the test set. The model parameters were tuned to optimize their performance (minimum RMSE) for each task. The RMSE is measured in the same units as the observed/predicted values and is severely affected by large error values. The coefficient of determination (R-squared), which evaluates how much of the output variance can be explained by the variation in the input variables, was also obtained. The goal was to maximize it and minimize the RMSE. The best predictive ML models obtained after training/cross-validation (minimum RMSE) or by the least square method, in the case of MLR, were further tested and compared against the same test set. The RMSE and R-squared values for the test sets evaluated the model performance working as a comparison tool to choose the best model.

## Figures and Tables

**Table 1:** Arrangement of *Aspergillus* spp. and *P. verrucosum* isolates in homogeneous groups, in terms of their overall susceptibility to the tested LAB strains, according to Duncan’s multiple range test (α = 0.05).

Fungal Species	Homogeneous Groups ^1^					
	Lower ◄—Susceptibility—► Higher					
	A	B	C	D	E	F
*A. flavus*		X				
*A. parasiticus*		X				
*A. carbonarius*			X			
*A. niger*	X					
*A. welwitschiae*	X					
*A. steynii*					X	
*A. westerdijkiae*				X		
*P. verrucosum*						X

^1^ The susceptibility increased from group A to group F. There were no statistically significant differences (α = 0.05) between the fungi showing an X within the same column.

**Table 2:** Arrangement of LAB strains in homogeneous groups, in terms of their overall efficacy in inhibiting fungal growth of the assayed fungi, according to Duncan’s multiple range test (α = 0.05). For LAB abbreviations, see the legend of Figure 1 and the Abbreviation list of the LAB species.

LAB Species (Strain)	Homogeneous Groups ^1^							
	Lower ◄—Efficacy—► Higher							
	A	B	C	D	E	F	G	H
Pp (M9MM5b)							X	
Pp (S11sMM1)								X
Pp (S1M4)				X				
Lmm (M8MG2)				X				
Lmm (T3Y6b)			X					
Lmd (T2MM3)						X		
Lpp (3T3R1)					X			
Lsc (T3MM1)	X							
Lsc (T3Y2)	X							
Cf (T3Y6c)					X	X		
Lb (M5MA4)		X						

^1^ The order of efficacy increases from group A to group H. There are no statistically significant differences between the strains showing an X within the same column. The presence of more than one X in the same row indicates overlap.

**Table 3:** Arrangement of LAB strains in homogeneous groups, in terms of their degree of efficacy to inhibit the growth of toxigenic *Aspergillus* spp. and *P. verrucosum*, according to *post-hoc* Duncan’s test (α = 0.05) ^1^.

LAB Strain	Fungal Species							
	*A. flavus*	*A.* *parasiticus*	*A.* *carbonarius*	*A.* *niger*	*A.* *welwitschiae*	*A.* *steynii*	*A.* *westerdijkiae*	*P.* *verrucosum*
Pp (M9MM5b)	1	7	8	7	5	3	6	6
Pp (S11sMM1)	6	9	7	7	7	3, 4	4, 5	6
Pp (S1M4)	5	4, 5	2	4	3	4, 5	2, 3	4
Lmm (M8MG2)	2, 3	6	4	3	3	2	5	3
Lmm (T3Y6b)	2	8	5	5	4	3, 4	5	1
Lmd (T2MM3)	6	5	6	3	3	5, 6	6	4
Lpp (3T3R1)	3	2, 3	3	1	2	6	7	5
Lsc (T3MM1)	2, 3	2	1	2	2	1	3, 4	2
Lsc (T3Y2)	2, 3	3, 4	1	1, 2	2	1	3, 4	2
Cf (T3Y6c)	2, 3	4, 5	6, 7	6	6	3, 4, 5	2	5
Lb (M5MA4)	4	1	5	2	1	1	1	4

^1^ Groups are denoted by a digit that increases with increasing efficacy. The presence of two or more digits separated by a comma indicates the overlapping of groups. Within a column, the LAB strains having the same digit(s) are not statistically different. For LAB abbreviations, see the legend of Figure 1 and the Abbreviation list of the LAB species.

**Table 4:** Best machine learning (ML) model performance for predicting the percentage of fungal growth inhibition (GIP) in dual cultures (MRS-CYA20S), based on all fungal isolates and LAB strains assayed and incubation temperature. The model performance was attained on the same test set.

ML Algorithm ^1^	Assayed Parameters	Best Model Parameters	RMSE ^2^	R-Squared
MLR	Regression coefficients	Found by the least-square method	0.2714	0.7629
MLP	*size*: 1–20; *decay*: 0.01, 0.05, 1.00	*size* = 7; *decay* = 0.01	0.1999	0.9232
RF	*mtry*: 2, 3; *ntry*: 500	*mtry* = 3	0.2268	0.8623
XGBoost	*max-depth*: 2–7; *eta*: 0.1–0.5); *subsample*: 0.5, 0.75, 1	*max_depth* = 5, *eta* = 0.2, *subsample* = 1	0.2828	0.7767

^1^ MLR: multiple linear regression; MLP: multilayer perceptron (neural network); RF: random forest; XGBoost: extreme gradient boosted trees (the following parameters of this algorithm had constant values: *nrounds* = 150, *gamma* = 0, *colsample_bytree* = 1, *min_child_weight* = 0.5). ^2^ RMSE: root mean square error.

**Table 5:** Retention time, limits of detection (LOD), limits of quantification (LOQ), mean recoveries (%), and mean relative standard deviations of recoveries (RSD) for mycotoxins analyzed by UPLC-MS/MS in dual solid medium (MRS-CYA20S).

Mycotoxin ^1^	Retention Time (min)	LOD (ng/g)	LOQ (ng/g)	Mean Recovery (%)	Mean RSD of Recoveries (%)
AFB1	8.30	0.78	2.34	85.2	9.5
AFB2	8.04	0.8	2.4	87.3	11
AFG1	7.80	1.18	3.5	83.0	8.2
AFG2	7.50	0.4	1.2	90.5	8.7
OTA	10.52	0.8	2.4	91.1	7.6

^1^ AFB1: aflatoxin B1; AFB2: aflatoxin B2; AFG1: aflatoxin G1; AFG2: aflatoxin G2; OTA: ochratoxin A.

**Table 6:** Ranges of mean reduction of aflatoxin concentration in cultures of *A. flavus* and *A. parasiticus* treated with LAB strains, compared to untreated controls.

		Fungi			
		*A. flavus*		*A. parasiticus*	
Mycotoxin	Temperature (°C)	Minimum Reduction (%)	Maximum Reduction (%)	Minimum Reduction (%)	Maximum Reduction (%)
AFB1	20	32.7	52.3	20.0	55.0
	25	22.8	37.9	19.7	44.7
	30	23.8	34.0	19.0	35.2
AFB2	20	32.9	57.0	20.7	55.3
	25	16.2	38.3	21.5	45.5
	30	20.6	34.9	21.4	60.8
AFG1	20	-	-	21.2	59.7
	25	-	-	22.4	44.4
	30	-	-	19.8	37.1
AFG2	20	-	-	25.0	59.7
	25	-	-	22.1	45.3
	30	-	-	18.6	35.5

**Table 7:** Arrangement of ochratoxigenic *Aspergillus* spp. and *P. verrucosum* isolates in homogeneous groups, regarding OTA production in cultures of the tested LAB strains, according to Duncan’s multiple range test (α = 0.05). OTA levels increased from group A to F.

Fungal Species	Homogeneous Groups					
	A	B	C	D	E	F
*A. carbonarius*				X		
*A. niger*			X			
*A. welwitschiae*		X				
*A. steynii*						X
*A. westerdijkiae*					X	
*P. verrucosum*	X					

**Table 8:** Ranges of mean reduction of ochratoxin A (OTA) concentration in cultures of ochratoxigenic fungi treated with LAB strains, compared to untreated controls.

Fungi	Temperature (°C)	OTA	
		Minimum Reduction (%)	Maximum Reduction (%)
*A. carbonarius*	20	22.6	55.6
	25	23.7	52.1
	30	22.1	48.4
*A. niger*	20	23.0	56.3
	25	27.0	37.0
	30	7.3	51.2
*A. welwitschiae*	20	28.8	77.9
	25	27.9	39.3
	30	21.1	87.4
*A. steynii*	20	44.5	94.8
	25	39.2	81.8
	30	33.5	75.6
*A. westerdijkiae*	20	18.7	61.1
	25	25.6	53.2
	30	23.8	57.3
*P. verrucosum*	20	36.7	100.0
	25	27.0	100.0
	30	35.6	100.0

**Table 9:** MS/MS conditions for the detection of mycotoxins.

Mycotoxin	ESI Polarity	Molecular Mass (Da)	Precursor Ion	*m/z* (Da)	Product Ion (*m/z*) (Da)	DP(V)	EP(V)	CE(V)	CXP(V)
AFB1	+	312.063	[M + H]^+^	313.1	285.2 ^1^	106	10	33	16
					128.1 ^2^	106	10	91	10
AFB2	+	314.079	[M + H]^+^	315.1	287.2 ^1^	96	10	37	18
					259.2 ^2^	96	10	43	18
AFG1	+	328.058	[M + H]^+^	329.1	243.1 ^1^	86	10	39	14
					200.0 ^2^	86	10	59	12
AFG2	+	330.074	[M + H]^+^	331.1	313.2 ^1^	111	10	35	18
					245.2 ^2^	111	10	43	14
OTA	+	403.082	[M + H]^+^	404.0	239.0 ^1^	91	10	37	16
					102.0 ^2^	91	10	105	14

DP: declustering potential; EP: Entrance potential; CE: Collision energy; CXP: Collision cell exit potential.^1^ Quantifier ion (q1); ^2^ Qualifier ion (q3).

## Data Availability

The data supporting reported results other than those included in Tables and Figures in the main text are available, on request, from the corresponding Author.

## Abbreviation List of the LAB Species

**Full Name**	**Abbreviation**
*Pediococcus pentosaceus*	Pp
*Leuconostoc mesenteroides* ssp. *mesenteroides*	Lmm
*Leuconostoc mesenteroides* ssp. *dextranicum*	Lmd
*Lacticaseibacillus paracasei* ssp. *paracasei*	Lpp
*Latilactobacillus sakei* ssp. *carnosus*	Lsc
*Companilactobacillus farciminis*	Cf
*Levilactobacillus brevis*	Lb
